# Cancer mortality inequalities in urban areas: a Bayesian small area analysis in Spanish cities

**DOI:** 10.1186/1476-072X-10-6

**Published:** 2011-01-13

**Authors:** Rosa Puigpinós-Riera, Marc Marí-Dell'Olmo, Mercè Gotsens, Carme Borrell, Gemma Serral, Carlos Ascaso, Montse Calvo, Antonio Daponte, Felicitas M Domínguez-Berjón, Santiago Esnaola, Ana Gandarillas, Gonzalo López-Abente, Carmen M Martos, Miguel A Martínez-Beneito, Agustín Montes-Martínez, Imanol Montoya, Andreu Nolasco, Isabel M Pasarín, Maica Rodríguez-Sanz, Marc Sáez, Pablo Sánchez-Villegas

**Affiliations:** 1 Servei de Sistemes d'Informació Sanitaria, Agència de Salut Pública de Barcelona, Barcelona, Spain; 2 CIBER Epidemiología y Salud Pública (CIBERESP), Parc de Recerca Biomédica de Barcelona, Barcelona, Spain; 3 Departament de Salut Pública, Facultat de Medicina, Universitat de Barcelona, Barcelona, Spain; 4 Universitat Pompeu Fabra, Barcelona, Spain; 5 Estudios e investigación Sanitaria, Departamento de Sanidad y Consumo. Gobierno Vasco, Vitoria-Gasteiz, Spain; 6 Observatorio de Salud y Medio Ambiente de Andalucía (OSMAN), Area de Salud Pública y Protección de la Salud, Escuela Andaluza de Salud Pública, Granada, Spain; 7 Servicio de Informes de Salud y Estudios, Instituto de Salud Pública, Dirección General de Salud Pública y Alimentación, Consejería de Sanidad, Comunidad de Madrid; 8 Servicio de Epidemiologia, Dirección General de Atención Primaria, Comunidad de Madrid; 9 Area de Epidemiología Ambiental y Cáncer, Centro Nacional de Epidemiología, Madrid, Spain; 10 Area de Desigualdades en Salud, Centro Superior de Investigación en Salud Pública de Valencia, Spain; 11 Instituto Aragonés de Ciencias de la Salud, Aragón, Spain; 12 Departamento de Medicina Preventiva e Saude Pública, Universidade de Santiago de Compostela, Spain; 13 Unitat d'Investigació en Anàlisi de la Mortalitat i Estadística Sanitaria, Departament d'Infermeria Comunitària, Medecina Preventiva i Salut Pública i Història de la Ciencia, Universitat d'Alacant, Spain; 14 Research Group on Statistics, Applied Economics and Health (GRECS), University of Girona, Spain

## Abstract

**Background:**

Intra-urban inequalities in mortality have been infrequently analysed in European contexts. The aim of the present study was to analyse patterns of cancer mortality and their relationship with socioeconomic deprivation in small areas in 11 Spanish cities.

**Methods:**

It is a cross-sectional ecological design using mortality data (years 1996-2003). Units of analysis were the census tracts. A deprivation index was calculated for each census tract. In order to control the variability in estimating the risk of dying we used Bayesian models. We present the RR of the census tract with the highest deprivation vs. the census tract with the lowest deprivation.

**Results:**

In the case of men, socioeconomic inequalities are observed in total cancer mortality in all cities, except in Castellon, Cordoba and Vigo, while Barcelona (RR = 1.53 95%CI 1.42-1.67), Madrid (RR = 1.57 95%CI 1.49-1.65) and Seville (RR = 1.53 95%CI 1.36-1.74) present the greatest inequalities. In general Barcelona and Madrid, present inequalities for most types of cancer. Among women for total cancer mortality, inequalities have only been found in Barcelona and Zaragoza. The excess number of cancer deaths due to socioeconomic deprivation was 16,413 for men and 1,142 for women.

**Conclusion:**

This study has analysed inequalities in cancer mortality in small areas of cities in Spain, not only relating this mortality with socioeconomic deprivation, but also calculating the excess mortality which may be attributed to such deprivation. This knowledge is particularly useful to determine which geographical areas in each city need intersectorial policies in order to promote a healthy environment.

## Introduction

Cancer has been considered a *modern *disease [1] due to its being linked with an increase in life expectancy. According to the study "The Global Burden of Diseases", 58.8 million people died during 2004, death being due to cancer in one eighth of them. It has been estimated that in 2008 there were 12.4 million new cancer cases, the majority of them in the Continent of America, West Pacific and Europe [2]. In Spain, cancer accounts for about a quarter of all deaths, e.g. 26.5% of all deaths in 2006 [3]. Despite the rise in incidence, cancer mortality is tending to decline in the European Union as a whole [4,5], as well as in Spain [3], something which has been attributed to early diagnosis and increased efficacy of treatments. In contrast, a rise has been detected in inequalities, both in between socioeconomic groups, and between countries and geographical areas [6-9].

Mortality observed in the population is influenced not only by individual-based factors or determinants, but also by contextual ones related to the environment in which one has lived [10]. These determinants are territorially unequally distributed generating unequal living conditions which end up affecting people's health. For this reason it is important to ascertain, through appropriate conceptual models, the population determinants of health, and the determinants of health inequalities [11]. In this sense, the political tradition and redistributive policies of countries are related with health and with mortality [12,13]. Spain is a country whose recent history presents a broad spectrum of sociopolitical change. In the recently inaugurated XXI century, an important part of this country's current population was born during a dictatorship, and lived through the political transition which led to the present modern democracy, with a health system providing universal coverage, and, among other changes, the country has evolved from being a country of emigrants to become a country which in the last decade has experienced an unprecedented, exponential rise in immigration [14]. All these political changes have involved changes in people's living conditions, and hence the importance of studying the inequalities experienced, while taking account of the environment.

Half of the world's population currently lives in large cities [15] and it is estimated that by 2030 the figure will reach 60% or more. Urbanization is usually linked with a country's economic growth and determines important changes in citizens' lifestyles, but does not necessarily imply improvement. Socioeconomic inequalities in health tend to be larger in urban areas with deprived and poor populations being concentrated in marginalized neighbourhoods and urban slums located at the centre or peripheral areas of these cities [16]. However, intra-urban inequalities in mortality have been infrequently analysed in European contexts. In recent years some studies have been conducted in Spanish cities which show a relationship between the socioeconomic deprivation of the geographical area of residence, and mortality [17-19]. But these studies have not focused on analysing inequalities in cancer mortality from a social and environmental perspective [20]. Thus, the aim of the present study was to analyse patterns of cancer mortality and their relationship with socioeconomic deprivation in small areas in 11 Spanish cities.

## Methods

### Design

Spain is located in southern Europe, and with more than 46 million inhabitants, is the fifth most populous country in the European Union. Administratively and politically it is organized into 17 autonomous communities, plus Ceuta and Melilla as autonomous cities.

This study was carried out in the framework of a project known as MEDEA *(Socioeconomic and environmental inequalities in mortality in small areas of Spanish cities - *http://www.proyectomedea.org/) conducted jointly by 10 Spanish research groups. The methodology of this study has been described elsewhere [21] , we here explain only the main aspects. The study uses a cross-sectional ecological design whose goal is to analyse mortality inequalities at the small area level in Spanish cities. Units of analysis were the census tracts of the eleven largest cities included in the study according to the 2001 Population and Households Census. These cities included 20.5% of the Spanish population in 2001 and are located in a variety of regions (Autonomous Communities) of Spain, from the wealthiest to the poorest: Catalonia (Barcelona), Comunidad de Madrid (Madrid), Euskadi (Bilbao), Aragón (Zaragoza), Comunitat Valenciana (Alicante, Castellón and Valencia), Galicia (Vigo), Andalucía (Córdoba, Málaga, Sevilla).

### Study population and information sources

The study population consisted of people residing in the cities during the period 1996 to 2003. Mortality data were obtained through the mortality registries of the Autonomous Communities or from the mortality registry of the city in the case of Barcelona.

The expected numbers of deaths in each census tract were calculated taking as reference the mortality rates by sex, age (5 year age mortality rates) and leading cause of death for Spain, year 2001, provided by the National Institute of Statistics (Instituto Nacional de Estadística). In order to elaborate an index of deprivation the source of data was the 2001 Population and Household Census. The Population and Household Census was also used to obtain information about the number of inhabitants stratified by sex, age (in five-year groups) and census tract.

### Mortality data

Number of deaths by five-year age group, sex, census tract of residence, and the underlying cancer cause of death were extracted from mortality registries. The census tract was obtained through the postal address of the deceased provided by either the Death Certificate or by the Local Census. Due to technical problems in geocoding place of residence, some deaths could not be geographically referenced, the proportions varying from 0.13% in Bilbao to 14.28% in Vigo. Except for Vigo, these percentages were always lower than 7%. Underlying cancer causes of death were coded using the International Classification of Diseases: 9^th ^revision (ICD-9) for deaths occurring between 1996 and 1998, and 10^th ^revision (ICD-10) for those occurring between 1999 and 2003. The groups of causes of cancer mortality studied and their ICD codes are shown in table 1.

**Table 1:** Population (number of inhabitants and census tract quartile distribution), number of census tracts and number of deaths by cause of cancer death.

	ICD-10	ICD-9	Alicante	Barcelona	Bilbao	Castellón	Córdoba	Madrid	Málaga	Sevilla	Valencia	Vigo	Zaragoza
**Population**													

Number of inhab			284,580	1,503,884	349,972	147,667	308,072	2,938,723	524,414	684,633	738,441	280,186	614,905

Quartiles in census tracts													

P_25_			931.25	746.00	895.00	1092.00	1053.50	952.00	962.25	990.25	862.25	962.00	1028.00

P_50_			1129.00	923.00	1188.50	1457.00	1330.50	1169.50	1180.50	1253.00	1135.00	1174.00	1276.50

P_75_			1336.75	1166.00	1493.75	1770.50	1621.25	1442.00	1457.00	1612.75	1460.50	1404.50	1566.00

**Number of census tracts**			222	1491	288	95	224	2358	422	510	598	236	462

**Causes of death in men**													

Stomach	C16	151	179	1051	353	101	140	2160	231	352	478	214	425

Colon	C18	153	259	1950	468	123	244	3116	315	659	781	240	583

Rectum	C19-C21	154	90	624	174	55	60	1082	99	162	258	86	287

Larynx	C32	161	69	522	209	54	99	989	148	224	228	59	211

Lung	C33-C34	162	882	5896	1282	447	805	9381	1335	2054	2513	722	2077

Prostate	C61	185	246	1864	465	178	197	3253	342	506	861	291	711

Bladder	C67	188	198	1228	266	86	189	1905	231	471	575	131	434

Hematologic	C81-C96	200-208	191	1471	282	90	197	2193	250	440	532	167	479

Total cancer*			3086	21493	5270	1576	2883	36417	4370	7106	9189	2937	7454

**Causes of death in women**													

Stomach	C16	151	104	813	206	64	86	1536	116	224	300	153	310

Colon	C18	153	237	1776	344	135	196	2678	303	596	687	190	495

Rectum	C19-C21	154	71	517	104	32	45	792	76	128	202	70	161

Lung	C33-C34	162	132	977	225	52	82	1533	146	216	339	116	249

Breast	C50	174	327	2539	525	136	316	3972	479	849	1036	280	797

Bladder	C67	188	46	327	60	19	35	452	62	76	92	39	96

Hematologic	C81-C96	200-208	165	1449	269	65	179	2109	254	433	509	175	409

Total cancer*			1906	14842	3241	883	1777	24380	2768	4737	5899	1919	4649

Men and women, 11 cities of Spain, 1996-2003

*All cancers, i.e. not just the types of cancer presented in the table.

### Socioeconomic deprivation index

A deprivation index was calculated for each census tract using the methodology proposed by Dominguez-Berjon et al [22] (principal component analysis) based on the socioeconomic indicators available for each census tract. Five simple indicators were included in this index (year 2001): a) Unemployment; b) Low educational level; c) Low educational level in young people (16-29 years); d) Manual workers; and e) Temporary workers. The index is normalized with a mean of 0 and standard deviation of 1, and the higher the index the higher the socioeconomic deprivation.

### Data analysis

It was assumed that the observed deaths for each census tract follow a Poisson distribution. In order to control the variability in estimating the risk or the Standardized Mortality Ratio (SMR), which is the ratio of observed and expected deaths in each census tract, we used Bayesian models, and more specifically the model proposed by Besag, York and Mollie (BYM) [23] which takes into account two types of random effects: spatial and heterogeneous. Prior distributions are assigned to the random effects, and hyper prior distributions to the parameters of the prior distributions. In this study, for the spatial effect, a conditional autoregressive normal distribution (CARN) was chosen. Following the suggestion made by several authors [24-26] a uniform distribution U (0,5) is assigned to the standard deviation of the random effects. In the model, the deprivation index was introduced as a quantitative variable.

As the scale of the deprivation index is adimensional, to illustrate the impact of deprivation on mortality we present, for every cancer cause of death and city, the Relative Risk (RR) of the census tract with percentile 95 of the deprivation index (highest deprivation) vs. the census tract with percentile 5 (lowest deprivation). This indicator can be considered a trimmed measure of the inequalities arising from deprivation for every city and cause, as it compares both ends of the scale and has been trimmed (to 5 and 95 percentiles) to make it more robust.

The estimations of RR were assessed through the mean of the posterior distribution and its 95% Credibility Interval (95%CI). This distribution was obtained using Monte Carlo methods based on Markov chains (MCMC), as implemented in the WINBUGS program, version 1.4.3 [27] and which was called from R 2.3.1 [28]. Model convergence was assessed using the *R-hat *statistic (Brooks-Gelman-Rubin statistic in WINBUGS) and effective sample size of the chains (n.eff statistic in R) [29]. Criteria for convergence were: *R-hat *less than 1.1 and *n.eff *greater than 100 for all the parameters summarized by the model.

In order to obtain the excess number of deaths related with socioeconomic deprivation we calculated the excess of deaths in each census tract comparing observed and expected deaths under the assumption that the deprivation of each area was the same as the average deprivation of the 10% of areas with the lowest deprivation [30,21]. The total excess of deaths was obtained by summing the excess deaths across all census tracts. We have also obtained the percentage of excess of deaths with respect to the total observed deaths. For each measure we have calculated its posterior mean and 95% posterior credibility interval.

All analyses were conducted separately for each city and for men and women [31]. The geographical distributions of smoothed SMR values derived from the BYM models are displayed using maps of septiles. The deprivation index is also displayed using a septile-based map. All maps were plotted using the R statistical package. All maps are presented in green and brown colours. Green colours show areas with lowest risk of mortality or lowest deprivation while brown colours show just the opposite.

## Results

Table 1 presents the number of inhabitants, according to the 2001 census, of each of the 11 cities included in the study, along with the number of census tracts into which it is divided, varying from 95 in Castellón with 147,667 inhabitants to the 2358 census tracts of Madrid, with nearly 3 million inhabitants. Considering all the cities, seventy-five percent of census tracts have populations of at most from 1166 people (Barcelona) to 1770 (Castellón). Finally, it also presents the number of deaths for the different types of cancer studied, the most common being lung, colon and prostate cancers among men, and breast, colon and lung cancers among women. Almost all the cities present the same pattern.

Table 2 and 3 show the associations between mortality and socio-economic deprivation for men and women respectively. Table 4 shows the excess of deaths due to socio-economic deprivation in both cases.

**Table 2:** Association between mortality by cancer in men and the socioeconomic deprivation index.

	Alicante	Barcelona	Bilbao	Castellón	Córdoba	Madrid	Málaga	Sevilla	Valencia	Vigo	Zaragoza
**Causes of death in men**	**RR 95% CI**	**RR 95% CI**	**RR 95% CI**	**RR 95% CI**	**RR 95% CI**	**RR 95% CI**	**RR 95% CI**	**RR 95% CI**	**RR 95% CI**	**RR 95% CI**	**RR 95% CI**

Stomach	1.95 1.63-3.08	1.71 1.30-2.17	1.73 1.22-2.38	1.52 0.63-2.90	0.73 0.35-1.35	1.67 1.44-1.94	1.52 0.93-2.41	1.35 0.90-1.91	1.44 1.07-1.96	1.05 0.55-1.74	1.33 0.96-1.83

Colon	1.28 0.77-2.03	1.10 0.90-1.35	1.18 0.80-1.66	1.31 0.62-2.31	0.88 0.51-1.44	1.06 0.92-1.24	1.01 0.66-1.53	0.87 0.66-1.14	1.06 0.82-1.35	0.63 0.38-0.93	1.23 0.91-1.62

Rectum	1.58 0.63-3.34	1.62 1.22-2.12	0.86 0.45-1.42	0.99 0.32-2.34	2.05 0.61-5.08	1.41 1.13-1.74	2.57 1.23-5.04	1.27 0.72-2.20	1.30 0.81-1.96	1.17 0.49-2.28	1.39 0.88-2.05

Larynx	3.77 1.43-8.20	3.51 2.64-4.63	3.36 2.10-5.09	3.35 0.82-9.93	3.25 1.51-6.18	2.86 2.20-3.57	2.84 1.54-4.82	3.79 2.41-5.65	1.66 1.04-2.54	1.44 0.53-3.18	2.95 1.80-4.69

Lung	1.83 1.41-2.35	1.90 1.66-2.15	1.76 1.44-2.14	0.97 0.65-1.37	1.36 1.00-1.78	1.91 1.73-2.09	1.80 1.39-2.34	1.88 1.54-2.26	1.51 1.26-1.80	1.03 0.71-1.43	1.48 1.25-1.75

Prostate	0.64 0.36-1.07	0.98 0.82-1.16	1.06 0.72-1.50	1.12 0.58-1.95	0.77 0.41-1.29	0.94 0.82-1.07	0.89 0.56-1.32	0.87 0.63-1.15	0.97 0.76-1.21	0.81 0.49-1.30	1.02 0.80-1.32

Bladder	1.18 0.58-2.07	1.61 1.29-2.00	1.43 0.86-2.22	1.03 0.38-2.32	1.37 0.72-2.28	1.36 1.15-1.63	1.47 0.94-2.26	1.41 0.99-2.03	1.14 0.85-1.48	0.82 0.40-1.43	1.11 0.78-1.56

Hematologic	1.29 0.69-2.23	1.13 0.92-1.38	0.71 0.43-1.09	1.04 0.39-2.21	0.38 0.20-0.65	1.06 0.89-1.25	0.77 0.47-1.19	1.02 0.68-1.47	0.84 1.22-1.53	0.92 0.49-1.63	1.16 0.84-1.56

Total cancer*	1.49 1.23-1.80	1.53 1.42-1.67	1.38 1.24-1.54	1.12 0.90-1.39	1.05 0.82-1.31	1.57 1.49-1.65	1.48 1.28-1.71	1.53 1.35-1.74	1.37 1.22-1.53	0.91 0.72-1.13	1.37 1.23-1.54

RR comparing percentiles 5 and 95 of the deprivation index. Men, 11 cities of Spain, 1996-2003.

RR: Relative risk of mortality 95% CI credibility interval

**Table 3:** Association between mortality by cancer in women and the socioeconomic deprivation index.

	Alicante	Barcelona	Bilbao	Castellón	Córdoba	Madrid	Málaga	Sevilla	Valencia	Vigo	Zaragoza
**Causes of death in women**	**RR 95% CI**	**RR 95% CI**	**RR 95% CI**	**RR 95% CI**	**RR 95% CI**	**RR 95% CI**	**RR 95% CI**	**RR 95% CI**	**RR 95% CI**	**RR 95% CI**	**RR 95% CI**

Stomach	0.68 0.26-1.44	1.74 1.34-2.20	1.56 0.82-2.61	1.25 0.45-2.69	1.00 0.41-2.04	1.51 1.26-1.77	1.26 0.54-2.37	1.14 0.70-1.74	1.71 1.11-2.54	0.92 0.46-1.57	1.25 0.85-1.76

Colon	1.06 0.59-1.76	1.00 0.84-1.18	0.85 0.54-1.25	0.94 0.45-1.67	0.48 0.24-0.84	0.87 0.75-0.99	1.05 0.67-1.57	0.94 0.69-1.25	1.13 0.83-1.46	0.59 0.35-0.96	0.98 0.71-1.28

Rectum	1.59 0.62-3.46	1.30 0.92-1.79	1.20 0.54-2.27	0.45 0.07-1.52	1.16 0.24-3.02	1.30 0.96-1.69	1.37 0.53-2.80	0.65 0.31-1.18	1.10 0.63-1.77	0.84 0.25-1.93	1.01 0.58-1.63

Lung	0.47 0.18-1.13	0.81 0.60-1.10	0.53 0.29-0.88	1.02 0.31-2.33	0.35 0.11-0.80	0.74 0.59-0.94	0.44 0.22-0.80	0.60 0.36-0.95	0.64 0.41-0.97	0.50 0.22-1.01	0.87 0.56-1.29

Breast	1.55 1.04-2.19	0.89 0.74-1.06	0.84 0.60-1.13	1.23 0.63-2.05	0.76 0.45-1.22	0.88 0.78-1.01	0.83 0.58-1.15	0.86 0.67-1.08	1.04 0.83-1.29	0.54 0.33-0.84	1.19 0.95-1.48

Bladder	1.93 0.60-5.06	1.25 0.81-1.85	0.62 0.17-1.47	0.87 0.09-3.77	0.58 0.10-1.68	0.94 0.67-1.29	1.81 0.67-3.93	1.45 0.62-2.98	1.22 0.53-2.33	0.76 0.21-2.11	1.03 0.46-1.86

Hematologic	1.68 0.96-2.80	1.05 0.87-1.25	1.18 0.75-1.81	0.85 0.24-2.02	1.03 0.56-1.79	0.98 0.83-1.15	1.18 0.75-1.76	1.04 0.75-1.42	1.09 0.80-1.43	0.64 0.36-1.06	0.71 0.51-0.98

Total cancer*	1.15 0.96-1.37	1.09 1.00-1.19	1.03 0.90-1.17	0.99 0.76-1.28	0.91 0.71-1.15	1.06 0.99-1.13	1.03 0.87-1.21	0.98 0.87-1.11	1.10 0.98-1.24	0.63 0.49-0.80	1.11 0.99-1.23

RR comparing percentiles 5 and 95 of the deprivation index. Women, 11 cities of Spain, 1996-2003.

RR: Relative risk of mortality

95% CI credibility interval

**Table 4:** Number of cases of cancer mortality and percentage of excess mortality under the assumption that deprivation of each area was the same as the average deprivation of the 10% of areas with the lowest deprivation.

	Men				Women			
**Cities**	**Number (total = 16.413)**	**% of excess**	**95% CI**		**Number (total = 1.142)**	**% of excess**	**95% CI**	

Alicante	481	15.57	6.65	23.75	108	5.61	-4.16	14.60

Barcelona	3736	17.38	14.15	20.83	502	3.38	-0.82	7.69

Bilbao	695	13.17	7.79	15.56	38	1.13	-6.31	8.09

Castellón	83	5.18	-8.53	16.92	-8	-1	-17.65	14.20

Córdoba	45	1.52	-10.53	12.57	-83	-4.73	-18.02	7.51

Madrid	7099	19.49	17.07	21.98	621	2.54	-0.89	6.05

Málaga	778	17.79	10.73	24.29	29	1.01	-8.83	9.79

Sevilla	1161	16.31	10.81	21.53	-48	-1.03	-7.14	5.23

Valencia	1343	14.61	8.53	20.00	260	4.40	-2.54	10.85

Vigo	-166	-5.68	-19.35	7.70	-508	-26.53	-44.43	-10.93

Zaragoza	1158	15.52	9.63	21.06	231	4.95	-1.39	11.05

Men and women, 11 cities in Spain, 1996-2003.

95%CI credibility interval

### Cancer mortality inequalities in men

**Figure 1:**
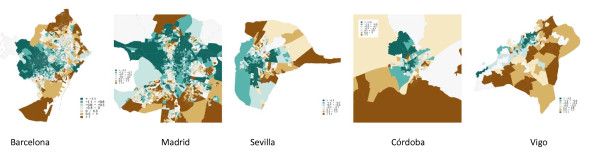
**Index of deprivation by census tract, in the cities of Barcelona, Madrid, Sevilla, Córdoba and Vigo**.

**Figure 2:**
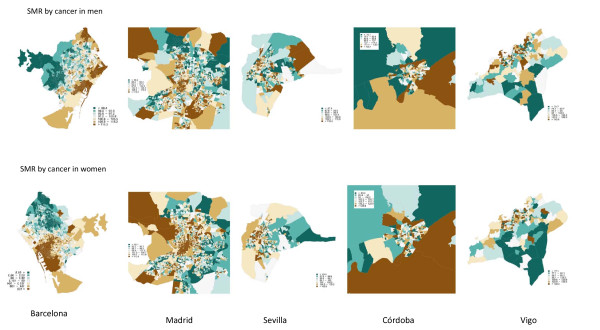
**Cancer mortality (smoothed Standardized Mortality Ratios) by census tract in men (top) and women (bottom) in Barcelona, Madrid, Sevilla, Córdoba and Vigo**.

In the case of men (table 2), socioeconomic inequalities are observed in total cancer mortality in all cities, except in Castellón, Córdoba and Vigo, while Barcelona (RR = 1.53 95%CI 1.42-1.67), Madrid (RR = 1.57 95%CI 1.49-1.65) and Sevilla (RR = 1.53 95%CI 1.36-1.74) present the greatest inequalities. The proportion of deaths due to cancer in these cities which may be attributed to socioeconomic deprivation (excess of deaths) would be, respectively, 17.38%, 19.49% and 16.31%. The excess number of cancer deaths due to socioeconomic deprivation was 16,413 (table 4). Figure 1 and 2 present, as an example, the maps for 5 cities, three with significant inequalities (Barcelona, Madrid, Seville) and two others (Córdoba and Vigo) with no inequalities. In Cordoba and Vigo we may observe a weak relationship between areas with socioeconomic deprivation and smoothed SMR, whereas in Madrid, Barcelona and Sevilla there are clear similarities between the spatial patterns of deprivation and of risk of mortality in many census tracts, although not in all.

In terms of type of cancer, lung and larynx present significant inequalities in all cities except Castellon and Vigo, with RR values above 3 in the case of larynx cancer in cities such as Alicante, Barcelona, Bilbao, Córdoba and Sevilla, this value being higher than the RR found for lung cancer. Other types of cancer which present significant inequalities in various cities are those of stomach, followed by rectum. In general Barcelona and Madrid, the two most populous cities in the country, present inequalities for most types of cancer, in particular both cities present inequalities for stomach, rectum, larynx, and lung cancer mortality. In contrast, in the smaller cities, Vigo and Castellón, no significant inequalities are found for any type of cancer (table 2).

### Cancer mortality inequalities in women

Among women (table 3) the pattern of socioeconomic inequalities differs from that of men. For total cancer mortality, inequalities have only been found in Barcelona and Zaragoza, cities in which the proportions of deaths attributable to deprivation are 3.38% and 4.95% respectively. In Castellón and Vigo, where no inequalities were found in men, the relationships for women are inverse and significant, with Vigo presenting an RR = 0.63 95%CI 0.49-0.80. Similarly, in Seville where men presented one of the highest levels of inequality, in the case of women the relation is inverse, although in this case not statistically significant.

Figure 1 and 2 present the distributions of socioeconomic deprivation and of cancer risk of mortality (smoothed SMR) among women in the same census tracts of the same cities as for men (Barcelona, Madrid, Seville, Cordoba and Vigo), and both the direct and inverse relationships, described above, may be seen. The case of Vigo stands out, where the majority of census tracts present this inverse relationship between deprivation and risk of mortality.

Stomach cancer mortality among women (table 3) presents significant inequalities in Barcelona (RR = 1.74 95%CI 1.34-2.20), Madrid (RR = 1.51 95%CI 1.26-1.77) and Valencia (RR = 1.71 95%CI 1.11-2.54). There is an important and significant inverse inequality with respect to lung cancer, which may be observed in 6 of the 11 cities under study: Bilbao, Cordoba, Madrid, Malaga, Seville and Valencia. Significant inverse inequality may also be observed for colon cancer in Córdoba (RR = 0.48 95%CI 0.24-0.84) and Vigo (RR = 0.59 95%CI 0.35-0.96). In the case of breast cancer, in general it presents an inverse relationship although this is only significant in Vigo (RR = 0.54 95%CI 0.33-0.84), while Alicante presents significant inequality, in the sense of higher levels of mortality in the more disadvantaged census tracts (RR = 1.55 95%CI 1.04-2.19). The inverse and significant relationship for hematological cancers in Zaragoza (RR = 0.71 95%CI 0.51-0.98) also stands out. The excess number of cancer deaths due to socioeconomic deprivation was 1142 (table 4).

## Discussion

The present study has detected, for the first time in Spanish cities, socioeconomic inequalities in total cancer mortality in men, whereas in women these inequalities disappear, and even there are cases of a pattern of an inverse relationship between area of residence socioeconomic deprivation and risk of mortality. The cities with the greatest inequalities are the country's largest cities, Barcelona and Madrid, but also Alicante and Seville. Moreover in the small cities, Castellón, Córdoba and Vigo, men present no inequalities, and women in Vigo present a significant inverse relationship. The pattern by cause of death among men showed that lung and larynx cancer had higher risk of mortality in areas with more socioeconomic deprivation in the majority of cites while among women lung cancer had an inverse relationship in six cities. The excess number of cancer deaths due to socioeconomic deprivation was 16,413 for men and 1142 for women.

### Interpretation of the results found

The various relationships found between deprivation of the area of residence and cancer mortality among men and among women are partly due to the important presence of the most common cancers in these two groups. Thus, in men the most common cancers are also the ones presenting the highest levels of inequality. In the case of women, breast and lung cancer mortalities in general present an inverse relationship with socioeconomic deprivation, as has previously been reported in the comparison of various European cities [32].

The results of the present study are in accordance with those found in these same cities when studying mortality due to various other causes, apart from cancer [21] and in the case of Barcelona, the inequalities described are also observed when studying the trends in inequalities over recent years, and which have a stable tendency to decrease [33].

In order to understand the influence of inequalities on cancer mortality, we must determine, among other things, the behaviour of the population in regard to the known risk factors linked to these diseases. In consequence, we have to acknowledge that cancer is related to smoking in many types of cancer (lung, mouth and pharynx, larynx, oesophagus, bladder, stomach, pancreas, and liver, among others) and the cause of 30% of deaths due to cancer worldwide. The consumption of alcoholic beverages is also associated with cancers of the mouth, larynx, oesophagus, liver, colon, rectum and breast in women. Finally diet, mainly linked to stomach cancer and to a considerably lesser extent to colon, breast and prostate cancers [1]. Even so, and although the main risk factors are known, many other environmental exposures are still to be identified, and are difficult to study.

The evolution of smoking has been different for men and women, depending on social class. In Spain, smoking in women basically affects the generations born since 1950. In Europe, over the last 50 years, smoking began among men, then spread to women, from North to South, and from the privileged social classes to the more disadvantaged ones [34,35], and hence cancer mortality related to this risk factor evolves in the same sense. Thus, it is observed that among women, in most cities, mortality reflects the greater presence of smoking in women of the highest socioeconomic levels [36,37] as they began smoking earlier. Currently, smoking is more common in women of the less privileged social classes, as has happened among men, so that it is to be expected that within a few years mortality due to causes directly related to tobacco, such as lung cancer, will be higher in women of the disadvantaged social classes.

Other contributing factors are also present, such as those of an occupational nature, particularly in the inequalities observed in lung and larynx cancer, since these are the most common among men in manual occupations and therefore in the areas of greater socioeconomic deprivation. Specifically, in the case of larynx cancer, between 20% and 30% of the inequality can be attributed to occupational exposures [38,39].

Stomach cancer also presents inequalities, in both men and women. It continues to be one of the most common cancer types worldwide, although the fall in prevalence of the main known risk factor in the developed countries, the Helicobacter pylori bacterium, has led to a decline in its presence [1]. Other factors such as dietary habits differ between men and women regardless of social level [40] and of socioeconomic level indicator used [41,42] which could partly explain the inequalities in stomach and colon cancers observed for men and for women. In the case of stomach cancer, it affects almost twice as many men compared to women, and this difference cannot be explained simply on the basis of different dietary habits. For this reason some authors suggest the possibility of an influence at hormonal level in the unequal presence of this cancer by gender [43,44].

There are also other factors associated differently between men and women, and within these, between social classes, which interact and may contribute to explain the observed results. Thus, leisure time physical activity is more common in the more privileged social classes [45]. Alcohol consumption also presents a differential pattern due to the influence of various sociocultural factors [46], as also happens with smoking. Smoking on its own, for example, does not increase the risk of breast cancer, whereas alcohol does, and combined with smoking this risk becomes more important [47].

All these highly inter-related health determinants cannot be isolated from the environments in which people live and work. Living in a city implies certain changes in lifestyle; in general urbanization has parallels with development, in the sense of having more opportunities, but these are not distributed equally over the city, and thus also for the social groups which live there. Thus, in all cities there are areas which could be considered healthy environments and others quite the contrary; moreover, in these areas the worst social and living conditions of the inhabitants are an obstacle to modify these conditions [48]. This implies the existence of risk factors characteristic of large urban nuclei and which must be taken into account, especially in ecological studies, such as for example atmospheric pollution or the worse job conditions of people living in more socioeconomically disadvantaged areas.

The important migratory movements occurring in cities all over the world, including Spain, mean there is a risk of generating important new pockets of poverty, apart from sociocultural alterations which affect the changes of aspect and personality of entire neighbourhoods, as is happening in certain areas of some Spanish cities [14]. It should be pointed out that although these changes have as yet had little effect on mortality, since the majority of the immigrant population is young, it is likely that this will change in the future.

### Limitations and strengths of the study

One limitation of the present study is the fact of aggregating information over different years, since this prevents us from having information about time trends [49]. Another limitation, is the bias that can have this kind of study due the unmeasured geographic mobility of the population [50], although we assumed that during these years mobility was not very important. Furthermore, during the period studied there was a change in the system for coding causes of death, ICD-10 superseding ICD-9, although one study conducted in various Autonomous Communities of Spain showed that there were no important differences in the classification of the leading causes of death, cancer among them [51].

The main contribution of the present study is that it presents, for the first time, inequalities in cancer mortality in small areas of various Spanish cities. The fact of describing mortality in small areas means that we obtain clearer "snapshots" of the spatial distributions of cancer mortality and of deprivation.

### Conclusions and recommendations

This study has described inequalities in cancer mortality in small areas of cities in Spain, not only relating this mortality with socioeconomic deprivation, but also calculating the excess mortality which may be attributed to such deprivation. This knowledge is particularly useful to determine which geographical areas in each city need intersectorial policies in order to promote a healthy environment [15,52].

## Authors' contributions

CB, RPR, AD, FDB, SE, AG, GLA, CMM, MAMB, AMM, AN, IMP, MS, PSV were all involved in designing the study. RPR wrote the first draft of the manuscript and bibliography review to which all authors subsequently contributed. MM, GS performed the statistical analysis and MG was involved in the elaboration of maps. MC, AD, FDB, SE, AG, CMM, MAMB, AMM, IM, AN, IMP, MRS, MS, PSV, CA were involved in collection of data and building the data bases. All authors made contribution to statistical analyses and interpretation of results, and revised the manuscript for important intellectual content. All authors read and approved the final manuscript.

## Competing interests

The authors declare that they have no competing interests.

## Note

This paper forms part of the PhD dissertation of Rosa Puigpinós I Riera in the Doctoral Programme in Public Health, University of Barcelona.
